# A Model for Visual Memory Encoding

**DOI:** 10.1371/journal.pone.0107761

**Published:** 2014-10-01

**Authors:** Rodolphe Nenert, Jane B. Allendorfer, Jerzy P. Szaflarski

**Affiliations:** 1 Department of Neurology, University of Alabama at Birmingham, Birmingham, Alabama, United States of America; 2 Department of Neurobiology, University of Alabama at Birmingham, Birmingham, Alabama, United States of America; 3 Department of Neurology, University of Cincinnati Academic Health Center, Cincinnati, Ohio, United States of America; Tokyo Metropolitan Institute of Medical Science, Japan

## Abstract

Memory encoding engages multiple concurrent and sequential processes. While the individual processes involved in successful encoding have been examined in many studies, a sequence of events and the importance of modules associated with memory encoding has not been established. For this reason, we sought to perform a comprehensive examination of the network for memory encoding using data driven methods and to determine the directionality of the information flow in order to build a viable model of visual memory encoding. Forty healthy controls ages 19–59 performed a visual scene encoding task. FMRI data were preprocessed using SPM8 and then processed using independent component analysis (ICA) with the reliability of the identified components confirmed using ICASSO as implemented in GIFT. The directionality of the information flow was examined using Granger causality analyses (GCA). All participants performed the fMRI task well above the chance level (>90% correct on both active and control conditions) and the post-fMRI testing recall revealed correct memory encoding at 86.33±5.83%. ICA identified involvement of components of five different networks in the process of memory encoding, and the GCA allowed for the directionality of the information flow to be assessed, from visual cortex via ventral stream to the attention network and then to the default mode network (DMN). Two additional networks involved in this process were the cerebellar and the auditory-insular network. This study provides evidence that successful visual memory encoding is dependent on multiple modules that are part of other networks that are only indirectly related to the main process. This model may help to identify the node(s) of the network that are affected by a specific disease processes and explain the presence of memory encoding difficulties in patients in whom focal or global network dysfunction exists.

## Introduction

Episodic memory is defined as the ability to consciously recall dated information and spatiotemporal relations from previous experiences, while semantic memory consists of stored information about features and attributes that define concepts [1], [2]. The visual encoding of a scene in order to remember and recognize it later (i.e., visual memory encoding) engages both episodic and semantic memory, and an efficient retrieval system is needed for later recall [3]. This entire process typically includes several important sequential and concurrent steps (e.g., visual attention, analysis of visual features and encoding of the scene features) that are crucial for it to be efficient and consistent.

The cortical underpinnings of these steps and processes have been examined in numerous neuroimaging studies. Primary visual features are encoded through a process of retinotopy [4], [5]. Then, a more precise categorization of visual information occurs via ventral and dorsal visual streams [6]–[9]. The capacity of the visual system to analyze a multi-object scene is limited [10]. Therefore, attentional mechanisms are needed to select relevant and filter out irrelevant information. Visual attention has been shown to improve the quality of visual encoding [11] by increasing contrast sensitivity [12], diminishing distractor's influence [13], and improving acuity [14]. Visual attention processes are widely distributed over the human cortex and appear to be controlled by networks located in frontal and parietal areas generating feedback information to the visual areas [11], [15]–[19].

A model for visual memory encoding based on human brain activity and functional connectivity during a scene-encoding task has not been developed to date. The aim of the present study was to build such a model using data-driven methods. In order to complete this task we used independent component analysis (ICA) of fMRI data combined with Granger causality algorithm (GCA). These advanced methods complement and add to the commonly used hypothesis-driven general linear modeling (GLM) method. While GLM permits identification of cortical and subcortical areas that constitute the underpinnings of the cognitive processes in question [20], it does not allow for the more detailed dissection and temporal arrangement of the individual components that potentially constitute the process to be examined, nor does it allow for the examination of the directionality of the information flow. Current theories agree that human higher cognitive functions emerge from a network of areas with precise interaction dynamics [21]. This is where the recently developed methods allow for in-depth analysis of the group fMRI data in order to uncover the processes that underlie visual memory encoding and sub-networks that support these processes. Further, these methods permit building a model for a specific cognitive process with that model later serving as the basis for examining the effects of a disease state on such a model e.g., epilepsy [22] and identifying nodes of the network that are specifically affected by the disease. The combined application of ICA and GCA to the analysis of blood oxygenation-level dependent (BOLD) data allows for the analysis of functionally connected cognitive networks and of the causal relations between them without required *a priori* information or preconceptions. Previously, the combination of ICA-GCA analyses has been successfully applied to various cognitive fMRI paradigms [23]–[28].

The first step in assessing the effects of disease states on cognitive networks is to build a robust model of the said network in healthy subjects so that these models can then be applied to testing and understanding of the cognitive deficits produced by the disease state. Recently, we have applied ICA to language fMRI data in order to build models for semantic decision, verb generation, and story processing [29]–[32], and we are currently testing the effects of stroke on such models. The aim of this study was to perform a comprehensive examination of the network for visual memory encoding using ICA and GCA of fMRI data to determine the directionality of the information flow and build a viable model of visual memory encoding that can serve as the basis for testing the effects of epilepsy (e.g., temporal vs. extra-temporal) on such a network.

## Methods

### Participants

Forty healthy controls (39% female) aged 19–59 (mean age = 33) with no history of neurological disorders or memory complaints were recruited. All subjects were included in our previous analyses of this task using GLM to provide functionally-defined fMRI regions of interest (ROIs) for the analyses of fMRI data collected in patients with epilepsy [22], [33]. This study was approved by the Institutional Review Boards (IRB) at the University of Cincinnati and the University of Alabama at Birmingham and all participants provided written informed consent prior to enrollment. Data sharing permission has been obtained from the IRB and the raw data are available, upon request from the authors.

### Functional MRI task

A block-design functional MRI scene encoding task was employed for the purpose of this study [33]–[35]. This task was used and described in our recent publication [22]. Briefly, during the active condition, participants were presented with stimuli that represented a balanced mixture of indoor (50%) and outdoor (50%) scenes that included both images of inanimate objects as well as pictures of people and faces with neutral expressions. Attention to the task was monitored by asking participants to indicate whether the scene was indoor or outdoor using a button box held in the right hand. Participants were also instructed to memorize all scenes for later memory testing. During the control condition, participants viewed pairs of scrambled images and were asked to indicate using the same button box whether both images in each pair were the same or not (50% of pairs contained the same images). Use of the control condition allowed for subtraction of visuo-perceptual, decision-making, and motor aspects of the task, with a goal of improved isolation of the memory encoding aspect of the active condition. Participants completed a practice run before entering the scanner in order to ensure full comprehension of the task. Practice items included five indoor/outdoor scenes as well as five scrambled pictures. Participants did not proceed to the scanner until they responded to all 10 images correctly. The paradigm included 14 alternating blocks of scrambled pictures (7 blocks) and scenes (7 blocks), starting with a block of scrambled pictures, for a total of 70 target pictures and 70 scrambled control pairs. The duration of the task was 7′15″. Each image was presented for 2.5 seconds, followed by a white blank screen for 0.5 seconds. Five whole brain volumes (15 seconds) were collected prior to initiating of the fMRI task run to allow for T2* equilibration – these volumes were discarded. Within 10–15 minutes of completing the scan, participants were administered a post-scan recognition test that included 60 indoor/outdoor scenes, with a balanced content of target and foil pictures. Foil pictures were chosen by matching contents and parameters of foil images to those presented in the scanner. Participants indicated whether they remembered seeing the picture in the scanner by pressing “Y” or “N” on a standard laptop keyboard (respectively “Yes” or “No”).

### Functional MRI

Images were collected on a 4-Tesla Varian MRI scanner. For each participant, an anatomical T1 scan was first collected (TR = 13 ms; TE = 6 ms; FOV = 25.6×19.2×15.0; flip angle array of 3: 22/90/180 with a voxel size of 1×1×1 mm). Manual shimming was performed next and was followed by a multi-echo reference scan (MERS) collected for correction of geometric distortion and ghosting artifacts that occur at high field (Schmithorst et al., 2001). Then, fMRI scanning was completed in thirty 4-mm thick contiguous planes sufficient to encompass the apex of the cerebrum to the inferior aspect of the cerebellum in the adult brain using the following echo planar imaging (EPI) protocol: TR/TE = 3000/25 ms, FOV = 25.6×25.6 cm, matrix = 64×64 pixels, slice thickness = 4 mm, flip angle array: 85/180/180/90. Task stimuli were delivered using Psyscope 1.125 [36] running on an Apple Macintosh G3 computer. Subjects were equipped with a button box to record responses and to alert the MRI technologist to any problems if necessary. Head movement was minimized with the use of foam padding and head restraints.

### Functional MRI data analysis

All imaging data were preprocessed and modeled using Matlab toolbox SPM8 (http://www.fil.ion.ucl.ac.uk/spm/software/spm8/). First, functional images were corrected for time discrepancy between slices (slice timing, interleaved mode, second to last slice as reference), corrected for motion (motion parameters were calculated with SPM8, normalized (EPI-weighted template, trilinear interpolation, 2×2×2 mm voxel size) and spatially smoothed with a 8-mm kernel full width half-maximum. The first 5 volumes were discarded from further analysis to allow for magnetic equilibration.

### General Linear Modeling (GLM) analysis

The fMRI data were initially processed using standard GLM methods to contrast active and control task conditions in single-subject analysis, while also covarying for head motion parameters and MR signal drift. In order to determine whether typical activations were obtained with this task, group-level analysis was performed using a one-sample t-test of the GLM results. The resultant group activation maps were comparable to the results of our previous studies [22], [33] and to the results of similar investigations from the literature [35], [37]. These analyses indicated typical pattern of BOLD signal changes when compared to these investigations (data not shown).

### Independent component analysis (ICA)

For the purpose of this study we adopted a previously developed group ICA method [38]. Group ICA is commonly used for making group inferences from fMRI data of multiple subjects. In our study, this was carried out using the Group ICA of fMRI Toolbox (GIFT; http://icatb.sourceforge.net) for not only estimating individual spatial patterns but also facilitating investigation of group differences under the same study condition. The individual datasets were temporally concatenated and reduced for computational feasibility through three stages of principal component analysis in order to reach the final dataset, which was then decomposed by ICA with Infomax algorithm into thirty-two spatially independent components [39]. Briefly, in GIFT, after each subject's functional data were reduced, the data were then concatenated into groups and put through another data reduction step. The number of subjects to put into each group is called partitions with the number of datasets in a partition being equal to the one-fourth of the number of data-sets selected for analyses (here N = 40 thus each partition had 10 datasets). After reduction within each partition the data were stacked into one group and put through the final data reduction. At this stage, the number of components was estimated using the minimum description length criteria [40], similarly to the common settings used in previous ICA-GCA studies [26]–[28], [41], [42]. The Infomax algorithm was repeated twenty times with randomly initialized decomposition matrices and the same convergence threshold using ICASSO approach in GIFT [43]. ICASSO allows for the estimation of small changes in the dataset as a result of changes in data stability; i.e., since the finite data never follows exactly the same ICA model, introducing ICASSO allows for estimating the reliability of the generated components [40], [43]. Subsequent to clustering of the obtained components, all centrotype-based components were selected and considered to be a stable result of the decomposition. Following back-reconstruction using GICA3 algorithm [44], components and their timecourses were averaged over all subjects. After careful visual inspection of the spatio-temporal characteristics of each identified independent component (IC), components reflecting noise were discarded [45]. In order to select components that have an active participation in visual memory processes, components of which time course showed a significant increase during control blocks compared to active blocks were discarded. Further analyses were conducted on the full time course of selected group-ICA components.

### Granger causality analysis (GCA)

Granger expressed the formal concept of causality for econometric purposes [46]. It is based on the common sense notion that causes imply effects during the future evolution of events and, conversely, the future cannot affect the past or present. By applying such considerations to temporal signals, if a time series “A” causes a time series “B”, then in some way knowledge about “A” should improve the prediction of “B”. More specifically, causality may be evaluated by comparing the variance of the residuals after an autoregressive (AR) application to the reference signal “A”, with the same variance being obtained when autoregression is evaluated on the past values of the signal “A” and the past values of the potentially causing signal “B”. GCA has been shown to be a viable technique for analyzing fMRI data [47]–[49] and to not vary after filtering [50]. Analysis of effective connectivity between the independent components was thus conducted using GCA, which models directional causality among multiple time series based on a variable autoregressive model [51]. The model order that represents the maximum time lag can be estimated using the Bayesian Information Criterion [52]. GCA was conducted by using a previously implemented MATLAB toolbox [53].

## Results

### Behavioral results

All subjects performed well above chance on the in-scanner scene and scrambled picture pair identification. Performance for the scrambled picture pair identification (control condition; 93.25±4.1%) was significantly better (p = 0.003) than for the indoor/outdoor scenes (active condition; 90.86±3%). All subjects also performed well above chance on the post-scan scene recognition task (86.33±5.83%).

### Independent component analysis

Thirty-two ICA components were identified. Of these, 10 were determined to be task-related (i.e., not representing noise or components related to the control condition) and were included in further analyses and model generation (Table 1). Each retained component was attributed to a particular network based on previously published data.

**Table 1:** Cortical localizations of the 10 task-related independents components: for each component we presented the anatomical location, corresponding Brodmann area(s), and the maximum Z-score with its Talairach coordinates (obtained using the Talairach utility provided in GIFT toolbox on group-ICA components maps).

Component ID	Area	Brodmann Area	Max Z-score (x, y, z) L/R
2	Superior Frontal Gyrus	6, 8, 9, 10	7.8 (−2, 59, 23)/8.7 (4, 56, 34)
	Medial Frontal Gyrus	6, 8, 9, 10	7.6 (−4, 56, 34)/7.9 (6, 52, 36)
	Anterior Cingulate	32	3.9 (−2, 47, 9)/3.7 (2, 45, 9)
	Middle Frontal Gyrus	8, 9, 10	3.1 (−22, 59, 21)/3.2 (22, 59, 21)
5	Cingulate Gyrus	23, 24, 31	6.9 (−2, −49, 28)/7.3 (2, −47, 28)
	Precuneus	7, 19, 23, 31, 39	6.7 (−2, −49, 32)/7.1 (2, −47, 32)
	Posterior Cingulate	23, 29, 30, 31	6.5 (−2, −49, 25)/7.0 (2, −47, 24)
	Cuneus	7, 18, 19	6.0 (0, −66, 33)/5.5 (4, −66, 31)
	Angular Gyrus	39	3.5 (−46, −66, 36)/3.1 (51, −63, 31)
	Inferior Parietal Lobule	39, 40	3.3 (−46, −64, 40)/3.0 (50, −60, 38)
	Supramarginal Gyrus	40	2.9 (−51, −59, 32)/3.3 (53, −59, 31)
	Superior Temporal Gyrus	39	3.1 (−53, −61, 29)/3.2 (53, −59, 27)
	Middle Temporal Gyrus	39	3.2 (−50, −63, 29)/3.1 (53, −61, 23)
10	Middle Frontal Gyrus	6, 8, 9, 10, 46	7.3 (−50, 17, 29)/NA
	Inferior Frontal Gyrus	9, 10, 44, 45, 46	7.0 (−50, 13, 29)/NA
	Precentral Gyrus	6, 9, 44	5.2 (−46, 19, 36)/NA
	Medial Frontal Gyrus	6, 8, 9	4.4 (−2, 39, 40)/3.9 (2, 39, 40)
	Superior Frontal Gyrus	6, 8, 9	4.1 (−30, 20, 52)/3.5 (2, 35, 46)
	Inferior Parietal Lobule	7, 39, 40	3.2 (−46, −56, 43)/NA
	Precuneus	19, 39	3.0 (−40, −70, 42)/NA
	Angular Gyrus	39	2.9 (−50, −61, 33)/NA
	Supramarginal Gyrus	*	2.8 (−51, −57, 30)/NA
	Superior Parietal Lobule	7	2.8 (−42, −58, 51)/NA
	Middle Temporal Gyrus	*	2.8 (−50, −61, 29)/NA
	Cingulate Gyrus	*	2.7 (−2, 23, 39)/NA
19	Inferior Frontal Gyrus	6, 9, 10, 44, 45, 46, 47	NA/5.6 (53, 19, 25)
	Middle Frontal Gyrus	6, 8, 9, 10, 11, 46, 47	NA/5.6 (51, 17, 32)
	Superior Frontal Gyrus	6, 8, 9, 10	NA/4.7 (34, 22, 50)
	Precentral Gyrus	6, 9, 44	NA/4.4 (46, 21, 36)
	Medial Frontal Gyrus	6, 8, 9	NA/3.4 (6, 31, 37)
	Inferior Parietal Lobule	7, 39, 40	NA/3.2 (50, −58, 40)
	Angular Gyrus	39	NA/3.1 (50, −58, 36)
	Precuneus	19, 39	NA/2.9 (40, −68, 38)
	Cingulate Gyrus	32	NA/2.9 (6, 23, 39)
	Supramarginal Gyrus	40	NA/2.8 (53, −57, 30)
20	Posterior Cingulate	23, 29, 30, 31	7.4 (−4, −60, 9)/7.4 (4, −62, 10)
	Culmen of Vermis	*	7.3 (0, −60, 1)/6.2 (4, −60, 0)
	Cuneus	7, 17, 18, 19, 23, 30	7.2 (−4, −64, 9)/7.0 (4, −64, 7)
	Culmen	*	7.1 (−2, −56, 1)/6.9 (2, −56, 1)
	Lingual Gyrus	18, 19	7.0 (−4, −64, 5)/5.9 (4, −68, 5)
	Precuneus	23, 31	6.7 (0, −69, 18)/6.4 (4, −61, 18)
	Cingulate Gyrus	31	3.5 (0, −59, 27)/3.0 (4, −61, 29)
	Parahippocampal Gyrus	30	NA/2.7 (12, −48, 4)
23	Lingual Gyrus	17, 18, 19	9.0 (0, −85, 3)/8.4 (4, −85, 3)
	Cuneus	17, 18, 19, 23, 30	8.6 (−2, −87, 6)/7.6 (4, −83, 6)
	Declive	*	6.6 (−4, −80, −11)/6.2 (6, −80, −11)
	Declive of Vermis	*	5.1 (−2, −74, −11)/5.1 (2, −74, −10)
	Middle Occipital Gyrus	18	4.6 (−10, −91, 14)/4.0 (10, −91, 16)
	Culmen	*	4.4 (−10, −68, −8)/3.6 (12, −68, −8)
	Fusiform Gyrus	19	4.1 (−20, −80, −11)/3.0 (22, −82, −13)
24	Postcentral Gyrus	1, 2, 4, 5, 7	6.1 (−4, −51, 67)/5.3 (6, −49, 65)
	Precuneus	7	5.2 (−2, −55, 60)/5.8 (4, −59, 60)
	Paracentral Lobule	4, 5, 6, 31	4.4 (−2, −44, 54)/4.7 (2, −42, 54)
	Superior Parietal Lobule	7	4.2 (−6, −63, 57)/3.8 (10, −65, 57)
	Medial Frontal Gyrus	6	3.0 (−4, −18, 67)/4.0 (4, −10, 67)
	Precentral Gyrus	4, 6	3.8 (−32, −22, 67)/2.6 (36, −20, 67)
	Inferior Parietal Lobule	40	3.2 (−46, −44, 57)/NA
	Superior Frontal Gyrus	*	2.8 (−30, −6, 65)/NA
	Thalamus	*	2.8 (−4, −5, 9)/2.8 (4, −5, 9)
	Anterior Cingulate	*	NA/2.7 (2, 11, 25)
29	Culmen	*	8.1 (−22, −49, −11)/9.6 (24, −51, −11)
	Declive	*	8.4 (−24, −53, −11)/8.8 (24, −55, −12)
	Fusiform Gyrus	19, 20, 37	7.3 (−22, −53, −7)/8.4 (24, −55, −9)
	Parahippocampal Gyrus	19, 30, 36, 37	5.9 (−26, −45, −10)/7.2 (26, −47, −8)
	Lingual Gyrus	18, 19, 30	3.3 (−28, −60, −5)/4.9 (22, −59, −5)
30	Superior Temporal Gyrus	13, 21, 22, 38, 41	5.7 (−46, −12, −6)/5.7 (46, −16, −6)
	Insula	13, 22	5.3 (−42, −16, −6)/5.5 (44, −12, −6)
	Middle Temporal Gyrus	21, 22, 38	5.0 (−50, −16, −6)/4.8 (50, −20, −4)
	Claustrum	*	4.2 (−38, −23, 1)/3.7 (36, −14, −4)
	Superior Frontal Gyrus	6, 8	3.0 (−8, 41, 50)/3.1 (4, 39, 51)
	Lentiform Nucleus	*	2.9 (−32, −16, 1)/2.7 (32, −19, −1)
	Caudate	*	2.8 (−34, −27, −4)/2.6 (34, −25, −4)
31	Culmen	*	16.0 (0, −47, −9)/15.0 (4, −47, −9)
	Cerebellar Lingual	*	15.3 (0, −43, −10)/14.4 (4, −43, −10)
	Declive	*	11.0 (0, −55, −12)/9.7 (4, −55, −12)
	Culmen of Vermis	*	9.6 (0, −63, −9)/7.9 (4, −62, −5)
	Declive of Vermis	*	5.8 (0, −71, −12)/3.9 (0, −69, −15)
	Lingual Gyrus	18, 19	3.8 (4, −74, −6)/3.3 (16, −60, −5)
	Fusiform Gyrus	19, 37	3.6 (−24, −49, −9)/3.9 (24, −51, −9)
	Parahippocampal Gyrus	19, 36, 37	3.4 (−24, −45, −10)/3.7 (24, −47, −9)

### Granger causality analysis

**Figure 1:**
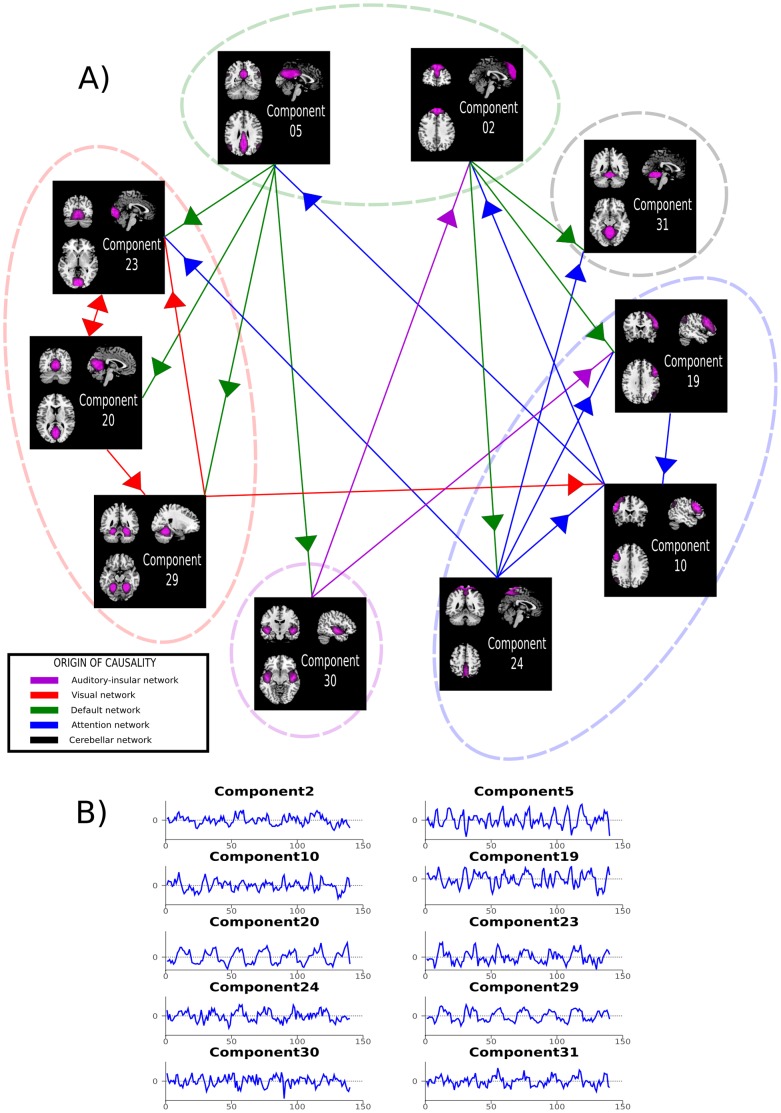
A) Relations and directionality of the information flow between task-related ICs. Details regarding each component are provided in Table 1. Each component was attributed to a particular network (See discussion section for a precise analysis). Each arrow is indicating a significant (p<0.05, FDR corrected) causal relation between two components. Component representations are in neurological convention (left hemisphere is on the left side of the image). B) Respective timecourse of components depicted in A)

**Figure 2:**
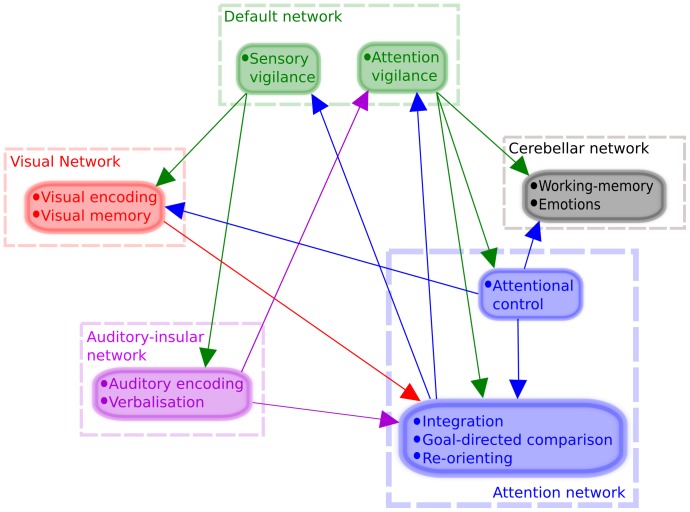
Proposed model for visual memory encoding based on results obtained in **Figure 1**. A precise description of the model is provided in the Discussion section.

Significant causality relations between each of the ten components (p<0.05; corrected for FDR) have been observed (Figure 1). These components are grouped into five networks that take part in the process of visual memory encoding: auditory, visual, default mode, attention, and cerebellar. Based on these networks a model of memory encoding is created, and the relative contributions of each of the specific networks are depicted in Figure 2. Below, we provide a description of each of the components with their potential implication for the scene-encoding memory network (Figure 1), the results of the causality analyses, and the construction of a visual memory model based on those results.

It should be kept in mind that GCA only provides information about causality between two events. Inferences made below about the temporal relationship between multiple events based on GCA results add to the current state of knowledge about cognitive functions related to the task performed by subjects. But, no algorithm that shows the statistical significance of such inferences is provided (see Discussion section).

### Independent components of the default mode network

Two ICs (IC 02 and IC 05) were identified that are typically shown to be activated when subjects are at rest and relaxed (Figure 1) [54]. But, in this study, these components are task-positive i.e., the activation in these areas occurred while the patients were performing the task at hand. Although seemingly counterintuitive, the default mode network that is activated here has been described to be involved in maintaining vigilance and it may be responsible for preparation for the new stimulus that is expected to come [55] and/or is involved in the modulation of the level of attention [56].

IC 02 is the superior frontal and anterior cingulate component of the default mode network that was previously found to participate in stimulus-oriented attention [57]. Further, this area has been linked to working memory and episodic memory encoding [58]. The IC 05 is the posterior/retrosplenial component of the default mode network that has been suggested to be an important node for information integration [59]. It was also previously shown to be involved in arousal and awareness [60], [61], controlling balance between internal and external attention [62], and in detection of environmental changes [55], [63], [64]. It was recently suggested that the posterior cingulate cortex also participates in a system that regulates the attentional focus [56].

### Independent components of the attention network

Three of the identified components appear to be a part of the network responsible for maintenance of attention. These components include ICs 10, 19, and 24 (Figure 1). The components 10 and 19 need to be considered at the same time as they are thought to constitute the fronto-parietal attention network [65]. The hemispheric temporal divergence between those two components suggests differences in cognitive functions within the same network. It has been suggested that the left and right frontal lobes have different involvement in the encoding and retrieval process with the left responsible for retrieval of semantic memory and simultaneous encoding of novel information into episodic memory while the right prefrontal regions are involved in the process of episodic memory retrieval (hemispheric encoding/retrieval asymmetry or ‘HERA’ model) [66]. Thus, participation in the attention network but somewhat different timing of that participation is easily explained when the HERA model of memory is taken into account. Further, in the right hemisphere, these components have been linked to inhibition and attentional control [67], stimulus-driven reorienting and resetting task-relevant networks [68], and also selective attention and target detection [69]. The right inferior frontal cortex (rIFC) was shown to be critical for behavioral updating, as in a go/no-go task [70], [71]. Clinical studies have put this component forward as a strong candidate for cortical area responsible for cognitive control [72], [73]. This area was also implicated in maintaining attention [67], [74]–[76]. Further, some studies have identified bilateral inferior frontal junction (IFJ) in the detection of visual motion whereas color detection preferentially engaged right IFJ [18], [77]. Other studies also identified hemispheric differences in IFJ activity using visual stimuli in that different fronto-parietal regions were found to be involved in attention to motion versus color features [78], [79]. The right IFJ has also been suggested to be involved into the selection of behaviorally relevant stimulus features [80].

Finally IC 24 involves activation of the intra-parietal sulcus and its surrounding cortical areas that have been strongly implicated in many higher cognitive functions such as spatial orientating and re-orientating [81], [82], which are necessary for the performance of the fMRI task that involves rapid shifting between visual analysis of indoor and outdoor scenes and analysis of matched and unmatched scrambled pictures.

### Independent components of the visual network

Three specific components belonging to this network were identified: ICs 20, 23 and 29 (Figure 1). The identified components cover primary and secondary visual area (V1, V2 and V3). Due to the visual nature of this fMRI task the involvement of the primary and secondary visual cortices is expected as such involvement was previously seen in this and similar versions of the task (ICs 20 and 23) [34], [83]. Further, since the scenes and scrambled pictures were presented visually, the visual cortices are involved in the processing of this task first. The sequential involvement of these areas most likely reflects the differences in retinotopy between the polar part and the frontal part of the calcarine sulcus [4], [5]. IC 29 appears to be a part of the cortical network for vision; it was previously identified as belonging to the ventral/ventrolateral visual stream [84]. This cortical area was found to be activated in studies where participants had to perform a task of face vs. non-face recognition [85]. This component probably reflects the process of categorization of the visual stimulus and/or the differences in processing formed vs. unformed images.

### Independent component of the auditory-insular network

A single component, IC 30, was identified as part of the auditory-insular network. Anatomically, this component includes predominantly primary and association auditory cortices. The activation in this cortical area corresponds most strongly to action–execution–speech, cognition–language–speech, and perception–audition paradigms [84]. Further, this component also includes posterior insula. Recently, three distinct cytoarchitectonic areas were identified in the human posterior insula [86]. Thus, it appears reasonable to think that these subdivisions form the anatomical substrate of a diversified mosaic of structurally and functionally distinct cortical areas. This may explain why activations in the insula have been reported for virtually all cognitive, affective, and sensory paradigms tested in functional imaging studies and have also been implicated by research in nonhuman primates. However, the most reliable evidence of an involvement of the posterior insula has been received for studies investigating painful [87], somatosensory [88], auditory [89], and interoceptive stimuli [90], as well as motor and language paradigms [91]–[93]. Thus, it is not surprising to note the involvement of these cortical areas in the execution of the task that involves not only visual but also other cognitive processes of working memory, face and scene recognition, decision making, and working memory.

### Independent component of the cerebellar network

A single and fairly large superior cerebellar component, IC 31, was identified. Superior cerebellum, especially the midline cerebellum has been implicated in many specific processes including e.g., language, emotion processing or visual memory manipulation [94]–[96]. Because of the inclusion of scenes with people in them and of faces with neutral expressions, it is possible that subjects interpreted not only whether the individual was stationed indoors or outdoors but also focused on facial emotions. Previous studies have postulated cerebellum to be involved in emotional processing via the cerebellar-hypothalamic pathways [97], [98]. A recent meta-analysis of “cerebellar” studies documented the involvement of the vermis and superior cerebellar hemispheres in emotional processing and postulated that these activations may be related to decision-making process in the studies of emotions rather than emotional processing itself [99]. Further, as alluded to above, cerebellar involvement could be related to the process of working memory manipulation [94].

### Network for visual encoding of scenes

Taken together, several nodes from multiple cognitive networks take part in the process of visual scene encoding. This process, the participation of the various nodes, and the directionality of the relationships are depicted in Figure 2. It is clear that the information enters the cognitive process via occipital visual cortices (visually presented information). A visual stimulus is retinotopically encoded in the primary visual cortex [5], the participation of ICs 20 and 23 likely reflects this encoding through the bidirectional information exchange between the two components. The third component of the visual network, IC 29, is characteristic of the so-called ventral visual pathway, associated with object recognition and form representation [100]. This is important as the presence of this component explains the further passage of the visually presented information to the other parts of the network via occipito-temporal connections responsible for fine encoding and maintenance in visual working memory of a visual stimulus through feedforward and feedback connections [6]. The only causal direction of information flow is to IC 10, one of the attentional components of the network with several uni- or bidirectional connections within this network and later outflow connections to other components of network for visual scene encoding.

This attention network is subdivided into fronto-parietal nodes (ICs 10 and 19) and a parietal node (IC 24). Altogether, these three components have been suggested to reflect a network which emphasizes start-cue and error-related activity and may initiate and adapt control on a trial-by-trial basis [101]. Considering that within this network causal relations were found from IC 24 to both ICs 10 and 19, but none toward IC 24, we suggest that the parietal component (IC 24) is the one responsible for adapting attentional control. The fact that this component receives a causal influence only from the default mode network (IC 02) further strengthens this hypothesis. Therefore, we posit that the two other components play a role of integrating information and analyzing that information in a task-driven way (cue and error-related activity). Both of the fronto-parietal components indeed receive causal influence from sensory networks (visual and auditory). These findings are in agreement with the previously proposed HERA model for memory encoding and retrieval [66].

The left fronto-parietal component (IC 10) receives a causal influence from the visual ventral pathway network (IC 29). This causality link from the so-called “what” visual pathway to cortical regions highly involved in language is likely to reflect the verbalization of the visual stimulus [102]. Moreover, the right fronto-parietal node receives a causal influence from the auditory-insular network (IC 30) and has a causal influence the left fronto-parietal component (IC 10). Both of those links could also reflect an involvement in the verbalization of the visual stimulus. We have recently shown that right hemisphere regions encompassed by the right fronto-parietal component (IC 19) can influence intra- or extra-scanner behavioral performance in semantic processing [103].

Within the attentional network, the left fronto-parietal component is the only one to have a causal influence on both components of the default-mode network (ICs 02 and 05). After encoding a visual stimulus with visual features and semantic information, the left fronto-parietal component is likely “activating” the default mode network in order to get ready for the next stimulus to come. Although it has been shown that right fronto-parietal regions are involved in stimulus-driven reorienting and resetting of the task-relevant networks [68], it is possible that the flow of information corresponding to this reorienting process needs to go through left fronto-parietal regions. This hypothesis is in agreement with recent results showing that the left dorso-lateral prefrontal cortex plays a necessary role in the implementation of choice-induced preference change [104].

The default mode network and cerebellar network play very important functions in the process of scene encoding. It is likely that the default network is involved into maintaining a certain level of vigilance, preparing for a new stimulus to come [55], and modulating the level of attentional focus [56]. Our results document a binary role of the default mode network. The posterior component (IC 05) has a causal influence on sensory networks (visual and auditory) and the frontal component has a causal influence on the attention network and on the cerebellar network.

Within the framework of the present study, one can only speculate of the implication of the cerebellar component. This component does not have a causal influence on any other network described here. It is possible that this component is actively involved in several cognitive processes related to this task as described above.

Finally, the auditory-insular network receives causal influence from the parietal component of the default network akin to the visual network. This is consistent with the hypothesis that the default network is responsible for maintaining a certain level of vigilance in sensory areas [55]. The auditory network has a causal influence on the left fronto-parietal component, sending auditory information for spatial and verbal integration (see attention network paragraph in discussion).

## Discussion

In this study, ICA and GCA were used to build a model of visual memory encoding based on fMRI data obtained from healthy subjects performing a visual scene-encoding task. Such a model for visual memory encoding based on human brain activity and functional connectivity during a scene-encoding task has not been developed to date. Building the groundwork that can be used as a baseline for future investigation of the effects of disease states on such network is therefore essential. As depicted in Figure 1, several components partake in the process of encoding visually presented stimuli. The nodes responsible for this process in healthy subjects are parts of five different networks and include auditory, visual, default, attention, and cerebellar networks. We discussed the relative contributions of the components of these networks and the integration of these seemingly unrelated components into an interactive network responsible for the complex task of visual memory encoding above.

The final level data analysis utilized in this study – Granger causality analysis – is a statistical algorithm for assessing causal influences between two simultaneously recorded time series [105]. Thus, a caveat to the interpretation of results must be considered. It is important to note that this algorithm cannot assess the existence of a cascade of events, which would be solely speculative. Also, GCA is very sensitive to BOLD fluctuations and to the fact that BOLD response may differ in different cortical areas [106]. However, inferences can be made about the temporality of multiple events based on our current knowledge of brain cognitive functions. Thus, while the above caveat puts the results of the study into certain perspective, the results may be interpreted as a series of events that need to occur in order for the cognitive process to be conducted efficiently. Moreover, the aim of the present paper is to build of model of visual memory that could potentially be used in further analyses and be compared to results of analyses that utilize other data processing methods.

In summary, this study identified several components of the network responsible for scene encoding and evaluated the directionality of the information flow within the network in order to build a model for visual memory encoding. While not complete, the proposed model lays the groundwork for further exploration of the processes and connections that are important for the maintenance and correct functionality of this network and for the examination of effects of various disease processes that may affect the functionality of this network, e.g., epilepsy.
